# Epigenetic Regulation of Stromal and Immune Cells and Therapeutic Targets in the Tumor Microenvironment

**DOI:** 10.3390/biom15010071

**Published:** 2025-01-06

**Authors:** Kang Liu, Yue Li, Minmin Shen, Wei Xu, Shanshan Wu, Xinxin Yang, Bo Zhang, Nengming Lin

**Affiliations:** 1 College of Pharmaceutical Sciences, Hangzhou First People’s Hospital, Zhejiang Chinese Medical University, Hangzhou 311402, China; kangliu@zju.edu.cn (K.L.); 202321014011138@zcmu.edu.cn (Y.L.); 20231061@zcmu.edu.cn (M.S.); 202421014011151@zcmu.edu.cn (W.X.); 202211113911046@zcmu.edu.cn (S.W.); 202211113911052@zcmu.edu.cn (X.Y.); 2 Department of Clinical Pharmacology, Key Laboratory of Clinical Cancer Pharmacology and Toxicology Research of Zhejiang Province, Affiliated Hangzhou First People’s Hospital, School of Medicine, Westlake University, Hangzhou 310006, China; 3 Department of Drug Clinical Trial Institution, Huzhou Central Hospital, Huzhou 313000, China; 4 Westlake Laboratory of Life Sciences and Biomedicine of Zhejiang Province, Westlake University, Hangzhou 310024, China

**Keywords:** tumor microenvironment, epigenetic modification, epigenetic therapy, immune therapy

## Abstract

The tumor microenvironment (TME) plays a pivotal role in neoplastic initiation and progression. Epigenetic machinery, governing the expression of core oncogenes and tumor suppressor genes in transformed cells, significantly contributes to tumor development at both primary and distant sites. Recent studies have illuminated how epigenetic mechanisms integrate external cues and downstream signals, altering the phenotype of stromal cells and immune cells. This remolds the area surrounding tumor cells, ultimately fostering an immunosuppressive microenvironment. Therefore, correcting the TME by targeting the epigenetic modifications holds substantial promise for cancer treatment. This review synthesizes recent research that elucidates the impact of specific epigenetic regulations—ranging from DNA methylation to histone modifications and chromatin remodeling—on stromal and immune cells within the TME. Notably, we highlight their functional roles in either promoting or restricting tumor progression. We also discuss the potential applications of epigenetic agents for cancer treatment, envisaging their ability to normalize the ecosystem. This review aims to assist researchers in understanding the dynamic interplay between epigenetics and the TME, paving the way for better epigenetic therapy.

## 1. Background

Cancer stands as a significant global health threat and the primary cause of mortality. Despite being a systemic disease, cancer manifests as a multifaceted ecosystem functioning as a complex ensemble of cancer cells, non-cancerous cells, an extracellular matrix (ECM), and assorted soluble factors [1]. Among the diverse stromal lineages infiltrating the tumor are cancer-associated fibroblasts (CAFs), endothelial cells (ECs), pericytes, and various tissue-resident cell types, including adipocytes and stellate cells, etc. Immune cells within the TME encompass innate immune cells, including tumor-associated macrophages (TAMs), myeloid-derived suppressor cells (MDSCs), neutrophils, natural killer cells (NKs), and dendritic cells (DCs), as well as adaptive immune cells such as T lymphocytes and B lymphocytes [2]. These cellular components are increasingly acknowledged as critical players in cancer initiation and progression [3].

Tumorigenesis entails genetic mutations and epigenetic alterations [4]. Coined by Conrad Waddington, epigenetics denotes the transmission of heritable cellular phenotypes without DNA sequence changes. Regulators of epigenetic modifications are categorized as writers, readers, erasers, and remodelers [5]. The dysregulated expression or aberrant activity of these regulators often leads to tumor development and progression [6].

The interaction between tumor cells and other components within the TME fuels the malignant traits of cancer cells, such as uncontrolled proliferation, aggressive invasion, and metastasis [7]. Recent research has indicated that epigenetic disruptions alter the phenotype of immune and stromal cells, thereby reshaping the TME [8]. In this review, we aim to comprehensively outline epigenetic modifications within the solid tumor TME. Additionally, we underscore the therapeutic implications of targeting these epigenetic regulators within the context of TME remodeling.

## 2. Fundamental Epigenetic Modifications

**Figure 1:**
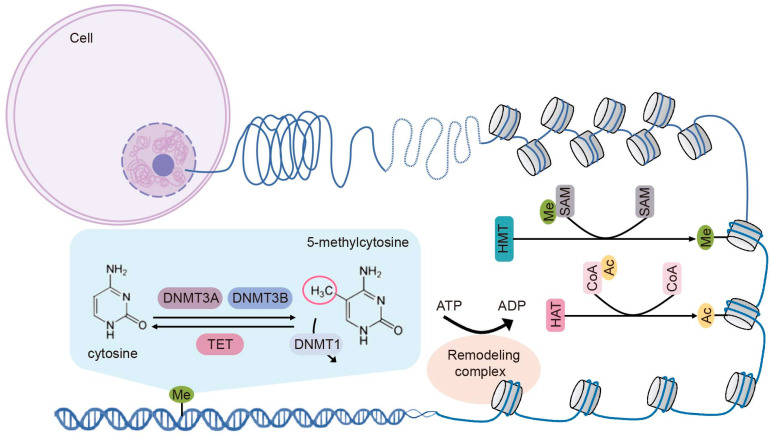
Schematic model of fundamental epigenetic regulation: DNA methylation, histone modifications, and chromatin remodeling. DNA methylation represents a dynamic process regulated by DNA methyltransferases, which transfer a methyl group to the cytosine residue. The methyl group can be actively removed from cytosines by TET enzymes. Histone modifications, including histone methylation and histone acetylation, are dynamic processes catalyzed by histone methyltransferases (HMTs) and histone acetyltransferases (HATs), respectively. The methyl and acetyl groups are added to the histone tails, resulting in chromatin configuration alterations. Chromatin remodeling is mediated by chromatin complexes that use the energy generated by ATP hydrolysis to eject, slide, and reposition nucleosomes, leading to the rearrangement of chromatin structure.

Global proteomic and genomic technologies have provided profound insights into the epigenomic alterations in cancer development, and the process of epigenetic modifications has been extensively reviewed [9,10,11]. Five key epigenetic mechanisms—DNA methylation, histone modifications, chromatin remodeling, RNA modifications, and non-coding RNA (ncRNA) alterations—govern the heritability of gene expression levels. Among these, ncRNAs and RNA modifications introduce significant functional changes to the transcriptome without altering the RNA ribonucleotide sequence, a focus that falls under the emerging field of epitranscriptomics [12,13]. Given that epitranscriptomics represents a distinct area extending beyond the scope of this review, we will center our attention on the classical epigenetic regulatory mechanisms: DNA methylation, histone modifications, and chromatin remodeling (Figure 1).

### 2.1. DNA Methylation

DNA methylation is an important epigenetic mark that orchestrates numerous biological processes, including gene expression, imprinting, chromosome stability, etc. [14]. It refers to the addition of a methyl group to the 5-carbon of cytosine residues (5mC) primarily within CpG islands located in gene promoters and regulatory elements. Methylated DNA assumes a closed state, impeding the binding of transcription factors and consequently suppressing gene expression [15].

DNA methylation is intricately regulated by distinct protein groups. The transfer of a methyl group from S-adenosyl-L-methionine to DNA’s cytosine residue is catalyzed by DNA methyltransferases (DNMTs), notably DNMT1, DNMT3A, DNMT3B, and DNMT3L. DNMT3A and DNMT3B, influenced by DNMT3L, create a methylation pattern on unmethylated DNA, while DNMT1 ensures the preservation of the DNA methylation pattern by methylating hemimethylated DNA during replication [16]. Methyl-binding proteins (MBPs) bind methylated CpG dinucleotides and interpret various DNA methylation statuses. MBPs consist of methyl-binding domain proteins, the Kaiso family proteins, and the SET and Ring finger-associated (SRA) domain proteins [17]. TET enzymes facilitate the active removal of methyl groups from cytosines. These enzymes convert 5mC to 5-hydroxymethyl-cytosine (5hmC), which can be further oxidized iteratively by TET proteins to 5-formylcytosine (5fC) and 5-carboxycytosine (5caC) [18]. In various cancer types, altered DNMTs, MBPs, and TET protein expression levels or functional mutations have been repeatedly reported [19].

### 2.2. Histone Modifications

Post-translational modifications of histones control the structure of chromatin, facilitating or hindering transcription factor binding and thereby orchestrating transcriptional activation and repression [20]. Various histone modifications have been discovered on the N-terminal tails of histone proteins, including acetylation, methylation, phosphorylation, ubiquitylation, SUMOylation, deamination, and lactylation, among others [21]. For brevity, we will discuss two major modes of histone modifications, methylation and acetylation, in the following sections.

#### 2.2.1. Histone Methylation

Histone methylation can occur on the side chains of lysine, arginine, and histidine residues [22]. Among various types of histone methylation, methylated modifications at lysine sites are best studied. This process is meticulously regulated by histone lysine methyltransferases (HKMTs) and lysine-specific demethylases (LSDs), acting collaboratively to add or remove specific methyl groups from critical target residues that regulate gene expression [23]. HKMTs exhibit remarkable substrate specificity for particular lysine residues and methylation states [24]. Histone demethylases are broadly categorized into two families, LSD and JMJD (JmjC domain-containing demethylase) [25]. LSD1 exclusively removes mono- and di-methylation modifications of H3K4 and H3K9, whereas the JMJD family targets lysine tri-methylation [26]. The outcomes of histone methylation, whether gene activation or repression, hinge on the site of methylation, the extent of modification, and the genomic context in which the modification exerts its influence [20].

#### 2.2.2. Histone Acetylation

Histone acetylation, catalyzed by histone acetyltransferases (HATs), involves adding acetyl groups to the ε-amino group of histone lysine residues. Three major families of HATs have been identified to date: the Gcn5-related N-acetyltransferase family (GNAT), the MYST family (MOZ, Ybf2, Sas2, TIP60), and the orphan family (CBP/EP300 and nuclear receptors) [27]. Acetylation of lysine residues is also a highly reversible process in which acetyl groups are removed by histone deacetylases (HDACs) [28,29]. Acetylated lysines carry a signal that is recognized by various “readers”, including the bromodomain proteins [30], which collaborate with HATs and HDACs to regulate gene expression.

### 2.3. Chromatin Remodeling

Covalent modifications of nucleosomes provide a dynamic platform for ATP-dependent chromatin structure remodeling. This DNA repackaging involves a set of specialized chromatin remodeling complexes, or remodelers, working in tandem with diverse factors that engage in chromatin assembly [31]. These remodelers are grouped into four distinct families: the switch/sucrose non-fermentable (SWI/SNF) family, the imitation switch (ISWI) family, the chromodomain helicase DNA-binding (CHD) family, and the inositol 80 (INO80) family [32]. By harnessing the energy generated by ATP hydrolysis, these complexes eject, slide, and reposition nucleosomes, consequently altering the structure and accessibility of chromatin DNA [33]. Moreover, remodelers could influence nucleosome stability and transcription initiation by mediating the exchange of the H2A/H2B dimers with histone variants [34].

## 3. Basic Cellular Components of the TME

The TME constitutes a diverse array of cells enveloped by secreted factors and extracellular matrix proteins, wielding a pivotal influence on tumor progression [35,36,37,38]. Prior to discussing the correlation between epigenetic alterations and the TME, we will first outline some fundamental cellular constituents that underpin the formation of the TME (Table 1). These include stromal cells, primarily cancer-associated fibroblasts (CAFs), and immune cells, specifically tumor-associated macrophages (TAMs), myeloid-derived suppressor cells (MDSCs), and tumor-infiltrating lymphocytes (TILs).

### 3.1. Cancer-Associated Fibroblasts

Cancer-associated fibroblasts (CAFs) are central constituents of the tumor stroma, significantly influencing the TME through extensive interactions with tumor cells and other cellular components [39]. CAFs primarily stem from the activation of resident fibroblasts or stellate cells in liver and pancreas cancer. However, emerging studies propose that in certain tumor types, CAFs can also arise from adipocytes, pericytes, endothelial cells, and bone marrow-derived mesenchymal stem cells [40]. Functionally, CAFs wield diverse mechanisms to regulate tumor growth and progression. For instance, CAFs directly enhance tumor cell survival and growth by secreting hepatocyte growth factor (HGF) and other growth factors [41]. Traditionally perceived as pro-tumoral, CAFs shape the immunosuppressive tumor microenvironment. However, in specific contexts, CAFs counteract tumor progression by promoting anticancer immunity and regulating tumor-inhibitory signaling [42]. The functions of CAFs, whether promoting or restraining tumor growth, are likely tied to their phenotypic heterogeneity and plasticity [43].

### 3.2. Tumor-Associated Macrophages

Tumor-associated macrophages (TAMs) constitute a significant proportion of immune cells infiltrating solid tumors, representing up to 50% of a tumor’s mass in certain tumor types [44]. TAMs are mostly originated from bone marrow-derived monocytes. Tumor cell-secreted chemokines, like CCL2, recruit bone marrow-derived monocytes and promote their differentiation in the tumor site. Nevertheless, emerging evidence highlights that the embryonic-derived tissue-resident macrophages could also serve as an alternative source of TAMs [45]. Functionally, macrophages exhibit two activation modes: classically activated M1 and alternatively activated M2 subtypes. M1-like macrophages elicit pro-inflammatory responses that favor an anti-tumor phenotype, while M2-like macrophages trigger anti-inflammatory responses conducive to an immunosuppressive milieu [46].

TAMs play a pivotal role in tumor progression, primarily as promoters and immunosuppressors. They drive angiogenesis by secreting angiogenic factors like vascular endothelial growth factor and chemokines, bolstering tumor progression. Moreover, TAMs contribute to the recruitment of T regulatory cells through the production of chemokines such as CCL2, CCL3, CCL4, CCL5, and CCL20. These chemokines dampen the activities of CD8^+^ T cells and NK cells, thereby forming an immunosuppressive microenvironment [47]. Clinically, TAMs in the TME are associated with poorer prognoses in various types of cancer, including breast, lung, and gastric cancers [48]. Moreover, TAMs are capable of communicating with a wide range of cell types within the TME, including cancer-associated fibroblasts, endothelial cells, and other immune cells. These extensive cell–cell interactions underpin their diverse functional roles [49].

### 3.3. Myeloid-Derived Suppressor Cells

Myeloid-derived suppressor cells (MDSCs) are a heterogeneous cell population with common myeloid origin in the TME [50]. MDSCs originate from the bone marrow and migrate to tumor locales, driven by chemokines emanating from tumor cells. There are two major types of MDSCs: polymorphonuclear MDSCs (PMN-MDSCs), which morphologically and phenotypically resemble neutrophils, and monocytic MDSCs (M-MDSCs), akin to monocytes [51]. MDSCs contribute to an immunosuppressive tumor microenvironment through diverse mechanisms. These encompass the obstruction of T cell trafficking, the upregulation of negative immune checkpoint molecules, and the generation of reactive oxygen and nitrogen species [52,53,54]. MDSCs also exert direct influence by producing immunosuppressive cytokines, such as IL-10 and TGF-β, to quell T lymphocyte function [55,56].

### 3.4. Tumor-Infiltrating Lymphocytes

Tumor-infiltrating lymphocytes (TILs) comprise diverse populations, including CD8^+^ T cells, CD4^+^ T cells, B cells, NK cells, and regulatory T cells (Tregs), all exerting significant impacts on tumor development and progression [67]. Notably, CD8^+^ T cells, particularly CD8^+^ cytotoxic T lymphocytes (CTLs), have long been regarded as chief immune effectors against cancer. CTLs recognize aberrant tumor-associated antigens displayed by class I peptide-major histocompatibility complex (MHC-I) on cancer cells through T cell receptors (TCR) [57]. Upon activation, CTLs eliminate cancer cells via granzyme and perforin-mediated apoptosis or FASL-FAS-induced cell death. The abundant infiltration of CTLs within the TME correlates with favorable prognoses in cancer patients [58].

CD4^+^ T cells exhibit a bipolar role in the TME. The Th1 subtype promotes the anti-tumoral response of cytotoxic CD8^+^ cells and B cells by secreting interferon γ (IFN-γ), interleukin-2 (IL-2), and tumor necrosis factor (TNF-α) [59]. Conversely, the Th2 subtype exerts a pro-tumoral effect through the secretion of anti-inflammatory factors [60]. Tregs, a subset of CD4^+^ T cells, predominantly curtail anti-tumoral immunity and expedite cancer progression through diverse mechanisms. Tregs support tumor cell survival both directly, by producing growth factors, and indirectly, through interactions with stromal cells such as CAFs and endothelial cells [61,62]. NK cells possess potent cytotoxic activity against tumors, serving as primary innate lymphoid cells that control tumor growth and metastasis [63,64]. B cells, a crucial component of the TME, predominantly drive the humoral immunity. Their contributions to anti-tumor immune response are gaining recognition [65,66].

The abundance of infiltrated immune cells classifies tumors into three subtypes: immune-infiltrated, immune-excluded, and immune-silent. While targeting other TILs, such as B cells, NK cells, and Tregs, can influence tumor growth and complement existing therapies, invigorating T lymphocytes within the tumor microenvironment remains the core focus of current immunotherapies [68]. Therefore, we will concentrate on the epigenetic changes in T lymphocytes in this review.

## 4. Epigenetic Modifications in TME

### 4.1. Epigenetic Modifications of CAFs

#### 4.1.1. DNA Methylation in CAFs

CAFs exhibit distinct phenotypes, gene expression profiles, and functions within the TME, despite a scarcity of genetic mutations. This suggests that the phenotypic plasticity of CAFs stems from underlying epigenetic modifications [69,70]. Multiple studies report that CAFs across various tumor types exhibit an identical genome-wide methylation pattern to that of tumor cells [71,72]. Within lung, gastric, and colorectal cancer, CAFs show overall DNA hypomethylation and targeted hypermethylation at specific genomic regions [73]. In contrast, Pidsley et al.’s work with CAFs from prostate cancer contradicts this trend, discovering no global hypomethylation using whole-genome bisulfite sequencing [74]. Recent studies have highlighted that normal fibroblasts undergo substantial DNA methylation changes upon transiting into CAFs. Specifically, one study identifies the hypomethylation and subsequent transcriptional activation of RUNX1 as the signaling nexus governing the epigenetic shift of tumor stroma [75]. These findings imply that the methylation pattern of CAFs may hinge on tumor type and stage.

DNA methylation regulates the conversion of stromal fibroblasts to CAFs [76]. Albrengues et al. reveal that a transient TGF-β stimulus induces a stable preinvasive phenotype on fibroblasts in the TME [77]. TGF-β triggers leukemia inducible factor (LIF) induction in both tumor cells and stromal cells, orchestrating the phenotypic shift of normal fibroblasts [77]. Notably, LIF prompts the hypermethylation of the protein phosphatase regulator SHP-1 at its promoter region, driven by increased DNMT3B expression [77]. Hypermethylation represses SHP-1 expression, leading to the activation of the JAK1/STAT3 signaling pathway, which is sustained by DNMT1 [78]. This activation prompts the reprogramming of stroma fibroblasts into pro-invasive CAFs [78]. Interestingly, cancer cells trigger DNMT1 activation, remolding CAFs into pro-tumor phenotypes via *Socs1* gene methylation in pancreatic cancer [79]. Furthermore, CAFs exhibit greater glycolytic activity compared to normal fibroblasts [80]. The metabolic reprogramming involves DNA methylation alterations on key metabolic enzymes by unclassified mechanisms under chronic hypoxia. Thus, further investigations are required to understand the evolution of tissue-specific DNA methylation in CAFs and its contribution to cancer progression.

#### 4.1.2. Histone Modifications and Chromatin Remodeling in CAFs

Numerous studies highlight the significance of histone modifications and chromatin remodeling in shaping the functional properties of CAFs. For instance, Tyan et al. revealed an intriguing connection between the expression level of ADAMTS1 and the behavior of normal tissue-associated fibroblasts co-cultured with breast tumor cells. This interaction promotes the invasion of the engaged tumor cells. The elevated ADAMTS1 expression correlates with the loss of EZH2 binding from its promoter region, leading to reduced H3K27 methylation [81]. In ovarian cancer, the stromal expression of the NNMT protein was found to be correlated with metastasis. NNMT was proved to be indispensable for the pro-tumoral traits of CAFs, as silencing NNMT in CAFs curbed ovarian cancer cell invasion and metastasis in vivo. The introduction of NNMT expression into CAFs led to a reduction in S-adenosyl methionine (SAM), alongside diminished H3K4 and H3K27 methylation on key genes regulating cytokine secretion and ECM deposition [82]. Interestingly, it has been demonstrated that methionine restriction did not directly result in the alteration of H3K4 methylation in fibroblasts [83], suggesting that epigenetic reprogramming might operate independently from the metabolic switch in CAFs. Taken together, these studies underscore the role of histone methylation in regulating the tumor-promoting identity of CAFs.

Histone acetylation tightly intertwines with DNA methylation and has been shown to promote CAF activation and their tumor-promoting functions. Specifically, H3K27 acetylation along with its reader protein BRD4 exhibited significant enrichment within the promoter and enhancer regions of the *Saa1* gene in gastric cancer fibroblasts. This is necessary for the pro-metastasis capacity of CAFs, and importantly, BRD4 inhibitors reinstate the anti-tumor phenotypes of CAFs in an SAA1-dependent manner [84]. Likewise, elevated H3K27ac on the promoter regions of *Ctgf* and *Postn* was found in hepatic stellate cells that had undergone myofibroblastic activation. This epigenetic shift, induced by TGF-β1, is responsible for the pro-metastasis behavior of CAFs in murine models of lung, colorectal, and pancreatic cancer [85]. This process relies on canonical histone acetylation and noncanonical mechanisms mediated by p300 acetyltransferase. Similarly, colorectal cancer-derived HSPC111 was established to promote liver metastasis through hepatic stellate cell activation [86]. The uptake of HSPC111 causes acetyl-CoA accumulation, consequently fueling H3K27 acetylation in hepatic stellate cells. H3K27 acetylation elevates CXCL5 expression and secretion, reciprocally acting on cancer cells to amplify HSPC111 production and enhance liver metastasis [86].

The regulation of CAFs by chromatin remodeling complexes remains less explored. Increased SATB2 expression was detected in CAFs within endometrial cancer, correlating with the pro-invasion properties of these CAFs [87]. Furthermore, skin cancer stromal cells showed extensive chromatin remodeling when transcriptional repressors such as ATF3 and CBF1 were depleted, underpinning the growth-promoting effects of CAFs [88]. Tumoral expression of chromatin remodeler protein HMGA2 is extensively linked to cancer metastasis and therapeutic resistance [89]. Intriguingly, the stromal expression of the HMGA2 protein independently predicts poorer clinical outcomes in pancreatic cancer and ampullary adenocarcinoma patients [90], indicating that parallel chromatin remodeling processes by HMGA2 occur in both tumoral and stromal compartments of cancers. When overexpressed in prostatic stromal cells, HMGA2 increases the secretion of WNT ligands, contributing to hyperplasia and multifocal intraepithelial neoplasia lesions in mouse models [91]. These studies collectively support the critical role of chromatin remodelers in controlling the pro-tumor phenotypes of CAFs, although the precise underlying mechanisms warrant further investigation.

### 4.2. Epigenetic Modulations of TAMs

#### 4.2.1. DNA Methylation in TAMs

DNA methylation is essential for the functional reprogramming of macrophages within the TME. Studies have indicated that DNA methyltransferases, including DNMT3B and DNMT1, regulate the phenotypic shift of macrophages in an inflammation setting. For instance, Yang et al. revealed that DNMT3B functions as a negative regulator for M2-like macrophage polarization in obese mice [92]. The depletion of DNMT3B promotes the M2-like phenotype in macrophages by mitigating the promoter methylation of the *Pparγ1* gene, consequently elevating its mRNA expression [92]. Similarly, in another study, DNMT1-induced hypermethylation of the *Socs1* gene in macrophages treated with lipopolysaccharide (LPS) propels the shift toward an M1-like phenotype by intensifying the release of proinflammatory factors [93]. It would be interesting to examine whether these mechanisms could be adapted to modulate the polarization of tumor-associated macrophages. Selective DNA methylation patterns on metabolic genes were recently reported in M1-like macrophages interacting with pancreatic ductal adenocarcinoma tumor cells [94]. Moreover, these macrophages demonstrate a transition from the M1 to the M2 state and acquire properties that favor cancer progression.

Unsurprisingly, enzymes catalyzing DNA demethylation play a role in macrophage polarization. Research demonstrates that TET1 activity is required for producing pro-inflammatory cytokines like TNF-α and interleukin-6 (IL-6), crucial for sustaining the M1 macrophage polarization [95,96]. In contrast, TET2 deficiency results in deleterious NLRP3 inflammasome activation, consequently elevating IL-1β secretion from macrophages and accelerating atherosclerosis development [97]. Supporting this, Alyssa H. Cull et al. found that although LPS stimulation induced TET2 in bone marrow-derived macrophages, it actually restricted the expression levels of key inflammatory cytokines such as IL-1β and IL-6 [98]. TET2 upregulation was shown to recruit HDAC2 to the promoter region, repressing *Il-6* gene transcription through a DNA demethylation impendent mechanism [99], suggesting an anti-inflammatory role for TET2 in macrophage differentiation. In melanoma models, targeted TET2 deletion in myeloid lineage reduced the tumor burden and dampened the immunosuppressive function of TAMs [100]. Collectively, these studies suggest DNA methylation’s role in macrophage polarization, yet comprehending the precise mechanisms across diverse tumor microenvironments necessitates future investigation.

#### 4.2.2. Histone Modifications in TAMs

Histone modifications wield a significant impact on macrophage phenotype and function. For example, Kittan et al. identified the induction of H3K4 methyltransferase SMYD3 during M2-like macrophage differentiation, which consequently promoted the expression of M2 marker genes [101]. Similarly, in the tumor context, cancer cell-derived exosomes or phosphatidylserine prompts increased expression level of JMJD3 in macrophages, resulting in M2-like polarization of TAMs and thereby promoting cancer metastasis [102]. Furthermore, M2-polarized macrophages showed JMJD3-dependent transcriptional activation of lysyl oxidase (LOX), which contributes to extracellular matrix remodeling and cancer metastasis [103]. These findings underscore the involvement of histone methylation in the emergence of M2-like macrophages.

Alterations in histone methylation have also been implicated in reprogramming macrophages toward an anti-tumor M1-like phenotype. For example, Wang et al. demonstrated that H3K9 methyltransferase G9a promotes M1-like macrophage polarization through the negative regulation of the CD36 molecule [104]. Lysine-specific histone demethylase 1A, commonly known as LSD1, appears to exert dual effects on triggering M1 polarization in macrophages [105,106,107]. On one hand, various groups have suggested that the genetic or pharmacological inhibition of LSD1 may amplify the inflammation response of macrophages under diverse conditions [108,109]. Notably, in breast cancer, M1-polarized macrophages demonstrated reduced expression levels of LSD1 alongside nuclear REST corepressor 1 (CoREST1) and the zinc finger protein SNAIL [110]. Disrupting LSD1–CoREST complexes with phenelzine activated H3K4 and H3K9 methylation, culminating in the transcriptional activation of M1-like marker genes in vitro and in vivo. Moreover, a combination of LSD1 inhibitors and chemotherapeutic reagents stimulates an M1-like antitumor response, significantly reducing the tumor burden in murine breast cancer models [111]. On the other hand, LSD1 has been reported as essential to the pro-inflammatory effect and the M1-like phenotype of bone marrow-derived macrophages in multiple studies [112,113]. The underlying reasons for these seemingly contradictory findings remain elusive.

Histone acetylation finetunes macrophage functions by regulating the transcription of critical cytokines. Shinohara et al. found that the extracellular vesicles derived from colorectal cancer cells contained miR-145, which was taken up by the progenitor cells of TAMs [114]. miR-145 directly binds to the 3′ untranslated regions of histone deacetylase 11 (HDAC11) mRNA, suppressing HDAC11 expression in these cells. Consequently, histone 3 acetylation increases, resulting in the upregulation of IL-10 and downregulation of IL-12 p40. This epigenetic shift cooperatively promotes an M2-like phenotype in EV-conditioned macrophages [114]. Histone acetylation also influences M2-like polarization through another cytokine, IL-6. Wang et al. demonstrated that IL-6 sustains the pro-migrative and angiogenic properties of M2-like TAMs in lung cancer [115]. They also discovered that IL-6 expression was regulated by increased histone-3 acetylation, which directly arises from stabilized histone acetyltransferase p300 by increased deubiquitinating enzyme USP24 expression in M2 macrophages [115]. It is now well-recognized that the acetylation of histone proteins couples with macrophage metabolic activities [116,117,118,119]. As an example, shortly after the TLR4-induced initiation of an inflammatory response, macrophages exhibited escalated glycolytic and tricarboxylic acid cycle activities, yielding additional acetyl-coenzyme A. The surplus acetyl-CoA fuels a global increase in histone acetylation, thus augmenting the pro-inflammatory response of macrophages [119].

### 4.3. Epigenetic Modulations of MDSCs

#### 4.3.1. DNA Methylation in MDSCs

MDSCs are the main promoters of the immunosuppressive tumor microenvironment in cancer. Both immune-suppressive MDSCs and immune-stimulating dendritic cells (DCs) emerge from bone marrow common myeloid progenitor cells. A comparison of DNA methylation between differentiated MDSCs and DCs reveals that MDSCs exhibit a global DNA hypomethylation profile with localized hypermethylation gains in specific DNA regions [120]. This pattern closely mirrors the DNA methylation pattern of CAFs and cancer cells. This similarity led the same study to propose that elevated DNMT3A activity might underlie the MDSC-specific methylation pattern and connect to MDSC’s immunosuppressive capacity, particularly in ovarian cancer [120]. In another study, Alyssa et al. reported that the IL-6/STAT3/DNMT signaling axis maintains the DNA methylation pattern in MDSCs. The activation of this axis leads to the accumulation of MDSCs within TME by impairing TNFα/RIP1-dependent necroptosis that promotes MDSC survival. Importantly, the use of DNA methyltransferase inhibitor decitabine (DAC) in mouse models results in the reversal of MDSC accumulation and an upsurge in the infiltration of antigen-specific cytotoxic T lymphocytes [121]. Intriguingly, DNA methylation patterns vary even in distinct MDSC subsets. For instance, Sasidharan Nair et al. uncovered that an increased DNA methylation signature was significantly upregulated in infiltrating immature MDSCs, while PMN-MDSCs showed downregulated methylation in flow cytometry-sorted MDSC subpopulations of colorectal cancer [122]. These findings revealed the influential role of DNA methylation in shaping the lineage specification and immunosuppressive phenotype of MDSCs.

#### 4.3.2. Histone Modifications and Chromatin Remodeling in MDSCs

The impacts of histone methylation on MDSC differentiation and function are relatively less explored. Infiltrating MDSCs are characterized by the overproduction of inducible nitric oxide synthase (iNOS), contributing to the suppression of the anti-tumor immune response [123,124]. Notably, Redd et al. identified a link between histone methyltransferase SETD1B and iNOS expression in tumor-induced MDSCs. More precisely, within MDSCs, SETD1B is upregulated and increases the trimethylation of histone H3 lysine 4 (H3K4Me3) at the *nos2* promoter, consequently leading to the pathological overproduction of iNOS. Likewise, significantly enhanced H3K4 trimethylation was detected at the promoter region of the osteopontin (*Opn*) gene within monocytic MDSCs infiltrating pancreatic cancer [125]. WDR5 recognizes the methylated H3K4 mark and facilitates the dimethylation and trimethylation processes, ultimately activating the transcription of target genes [126]. In mouse models, the use of the WDR5 inhibitor effectively erased H3K4me3 at the *Opn* promoter, subsequently decreasing *Opn* gene expression levels. As a result, the immunosuppressive capacity of MDSCs diminished, further leading to reduced tumor growth and enhanced therapeutic efficacy of anti-PD-1 immunotherapy in pancreatic cancer models upon WDR5 inhibition. Undoubtedly, other histone methylation modulators that influence MDSCs accumulation and function could also synergize with immune blockade therapies for cancer treatment [126].

Numerous studies have highlighted the important role of histone acetylation in driving MDSC’s pro-tumoral properties. In colorectal cancer, genes related to histone deacetylase are increased in infiltrating immature MDSCs along with the concurrent upregulation of DNA methylation genes. This was coupled with a concomitant reduction in histone acetyltransferase activity in this MDSCs population. Further insight emerges from exploring distinct members of the histone deacetylase family, each exerting specific regulatory functions in MDSCs. For instance, Sahakian et al. reported the restraining effect of HDAC11 on the expansion and immunosuppressive role of MDSCs in the thymoma tumor-bearing mice model. HDAC11 depletion was required to steer myeloid precursor cells toward monocytic MDSCs, significantly enhancing the immunosuppressive trait of M-MDSCs [127]. In contrast, Youn et al. proposed that polymorphonuclear MDSCs (PMN-MDSCs) are the predominant MDSCs population responsible for immunosuppression [128]. They also pointed out the involvement of HDAC2 in the accumulation of PMN-MDSCs in the TME. Specifically, HDAC2 appeared to play a pivotal role in promoting M-MDSCs differentiation toward PMN-MDSC. This was achieved through the direct modification of histone acetylation on the *Rb1* promoter, resulting in the silencing of RB1 expression in diverse mouse models like 4T1 mammary carcinoma, Lewis lung carcinoma, and EL-4 thymoma [128]. Together, these studies indicate the significance of histone acetylation in modulating MDSC expansion and suppressive functions.

Limited independent research has explored the role of the chromatin remodeler in MDSCs. Among these, a notable contribution by Almeida Nagata et al. unveiled a link between chromatin remodeling complexes and histone acetylation in the regulation of MDSC differentiation and function [129]. In their study, the researchers demonstrated that the activity of CBP/EP300 bromodomain (BRD) dictated the H3K27 acetylation pattern on cis-elements of pro-tumoral genes in MDSCs. Notably, pharmacological inhibition of CBP/EP300 BRD induced H3K27 acetylation deposition and thereby reprogramed the suppressive MDSCs to become inflammatory. This phenotypic change relied on the inhibition of genes related to the STAT pathway, alongside immunosuppression mediators such as Arg1 and iNOS [129].

### 4.4. Epigenetic Modulations of TIL T Cells

#### 4.4.1. DNA Methylation in TIL T Cells

Alterations of DNA methylation patterns directly regulate TIL infiltration and function. Distinct methylomes have been identified for different subsets of tumor-infiltrating T cells through genome-wide methylation profiling [130]. Notably, reactive T cell populations simultaneously showed specific demethylated patterns on both tumor-reactive marker genes and exhaustion-associated genes, indicating that DNA methylation may be linked to T cell identity.

The trafficking of effector T cells into a tumor relies on the production of Th1-type chemokines, which is also critical for effective immunotherapy response. Peng et al. demonstrated that DNMT1-mediated DNA methylation, in coordination with EZH2-mediated H3K27 trimethylation, suppresses the level of Th1-type chemokines CXCL9 and CXCL10 in an ovarian cancer model [131]. The concurrent inhibition of EZH2 and DNMT1, but not individual agents, significantly boosts T cell infiltration, slows down tumor progression, and enhances the therapeutic response to the PD-L1 checkpoint blockade in tumor-bearing mice.

The infiltration of regulatory T cells is often linked to poorer responses to immunotherapies and other cancer treatments [132,133]. Tregs necessitate a certain level of *Foxp3* gene expression for development and phenotypic maintenance. Yang et al. [134] reported that TET1 and TET2 catalyze the conversion of 5mC to 5hmC in the *Foxp3* gene in a lineage-specific manner in Tregs, leading to *Foxp3* gene hypomethylation and functional Treg maintenance under immune homeostasis [134]. Similarly, reduced *Foxp3* gene methylation was observed in infiltrating Tregs, correlating with their abundance in liver cancer [135]. In mouse models, T cell-specific DNMT1 knockdown altered the methylation patterns of *Foxp3* promoter and CpG region and increased the number of infiltrating Tregs [135]. These studies collectively support the critical role of DNA methylation in T cell lineage specification and anti-tumor immune response. It is worth noting that the T cell-specific DNA methylation pattern can serve as a sensitive approach to evaluating long-term survival or immunotherapy benefits in cancer patients [136,137].

#### 4.4.2. Histone Modifications and Chromatin Remodeling in TIL T Cells

Aberrant methylating patterns of histones correlate with the impaired antitumoral immune responses of CD8^+^ T cells in tumors. For example, in a recent study, Bian et al. reported that tumor cells outcompete CD8^+^ T cells for methionine consumption, resulting in the loss of H3K79me2 marks in effector T cells [138]. This reduction in H3K79me2 levels led to downregulated STAT5 expression, subsequently impairing T cell immunity against cancer. Remarkably, methionine supplementation restored the defective H3K79me2–STAT5 axis in tumor-infiltrating CD8^+^ T cells and significantly boosted the immune checkpoint blockade therapy in mouse models with syngeneic tumors [138]. Histone methylation can also indirectly influence the function of CD8^+^ T cells. As discussed earlier, the study led by Peng et al. revealed that combined EZH2 inhibition with DNA methylation inhibition elicits the secretion of Th1-type chemokines in tumors, amplifying the immune response against cancer [131]. Moreover, another study from the same group found that knocking down polycomb repressive complex 2 (PRC2) components other than EZH2 or overexpressing the H3K27-specific demethylase JMJD3 significantly augments the production of Th1-type chemokines in the TME [139]. EZH2 inactivation has been shown to specifically enhance helper T cell differentiation by modifying H3K27me3 patterns at the promoters of *Tbx21* and *Gata3* genes [140]. SUV39H1, the histone methylase responsible for H3K9me3, has been shown to be crucial for Th2 lineage identity [141]. SUV39H1 deficiency leads to the removal of H3K9me3 marks at *Th1* gene promoters, reducing HP1α binding at the same loci. This event drives the re-expression of the *Th1* gene and supports the trans-differentiation of Th2 cells into Th1 cells [141]. Likewise, SETDB1 was demonstrated to regulate the lineage integrity of helper T cells. SETDB1 knockout removes H3K9me3 marks from a Th1-specific set of endogenous retroviruses, indirectly boosting Th1 gene expression in helper T cells and promoting the acquisition of a Th1 phenotype [142]. However, whether these findings can be generalized to tumor-infiltrating helper T cells and guide the optimization of current immunotherapy remains largely unexplored.

TILs often experience exhaustion due to prolonged exposure to tumor antigens, and this exhaustion can be attributed in part to acetylated modifications of histone proteins. For instance, RNA splicing factor SRSF2 was found to be upregulated in exhausted T cells [143]. The acetyl-transferase P300/CBP complex interacts with SRSF2, facilitating the repositioning of acetylated H3K27 marks near immune checkpoint genes. This rearrangement affects STAT3 recruitment to the promoters of these genes, ultimately leading to TIL exhaustion [143]. The fluctuations in cellular acetyl-CoA levels, the main substrate for histone acetyltransferases, influence histone acetylation, which in turn impacts TIL exhaustion. For example, in the low-glucose tumor microenvironment, acetate is an alternative carbon source for immune cells. Exhausted T cells uptake acetate and convert it into acetyl-CoA. Acetyl-CoA fuels histone acetylation at restimulation-associated genes, such as IFN-γ, leading to the re-expression of those effector genes and the revitalization of effector T cells [144]. Extracellular potassium can also influence cellular acetyl-CoA levels. Necrotic tumors release cellular contents, increasing the extracellular potassium concentration and thereby impeding TIL’s uptake of nutrients [145]. This nutrient restriction limits acetyl-CoA production in T cells, resulting in reduced histone acetylation in genes associated with effector T cells. Interestingly, rather than entering into an exhaustion phenotype, the authors concluded that the depletion of acetyl-CoA seems to reprogram T cells toward a stem cell-like phenotype. Likewise, a recent study suggested that CD8^+^ T cells preferentially utilize ketone bodies for the synthesis of acetyl-CoA [146]. Effector T cells exhibit a significant increase in ketolysis activity, which produces more acetyl-CoA. This surplus acetyl-CoA fuels histone acetylation on effector genes and is required for the optimal immune response of effector T cells [146].

Chromatin-remodelers also regulate TILs’ function. One example is the essential role of CHD4, a subunit of the NuRD complex, for T cell development [147]. The expression level of CHD4, also known as Mi-2β, is increased during T cell development. Notably, the conditional knockout of CHD4 impairs various facets of T cell differentiation, including β-selection, CD4 lineage specification, and the expansion of mature T cells. While the NuRD complex generally operates in transcriptional inactivation, CHD4 directly binds to the enhancer of the *Cd4* gene and enables *Cd4* gene expression in developing T cells. The binding of CHD4 facilitates the recruitment of the E-box binding protein HEB and histone acetyltransferase P300, ultimately creating a hyperacetylated histone H3 landscape in this enhancer region. Such a landscape is indispensable for CD4 expression and T cell lineage specification. It remains an intriguing question whether CHD4 functions here independently of the entire NuRD complex [147]. Moreover, a recent study led by Belk et al. systemically assessed the regulatory mechanisms of T cell exhaustion through genome-wide CRISPR/Cas9 screening and unexpectedly uncovered the enrichment of subunits of cBAF and the INO80 complex in this process [148]. Subsequent analysis suggested that depleting these chromatin remodeling factors, particularly ARID1A, significantly improves the anti-tumor immune response by decreasing exhaustion-related gene expression and increasing the effector gene expression [148].

## 5. Epigenetic Therapies Target TME

Epigenetic regulators and their modifications are frequently altered in cancers [149,150,151]. Unlike genetic mutations, these epigenetic changes can often be reversed as enzymes or chromatin-binding proteins responsible for these modifications are usually targetable. This makes epigenetic therapy an appealing approach against cancers [152,153]. There are various epigenetic therapies that have received FDA approval or are currently undergoing clinical trials for both hematological and solid tumors [154,155]. Epigenetic drugs, such as DNA methyltransferase inhibitors and histone modification inhibitors, hold promise for transforming the TME from immunosuppressive to antitumoral by influencing stromal and immune cells (Table 2). Here, we will discuss the prominent mechanisms through which epigenetic drugs impact these cells in the TME.

### 5.1. DNA Methyltransferase Inhibitor

DNA methyltransferase inhibitors (DNMTi), such as 5-azacitidine (5-Aza), decitabine, eugenol, and guadecitabine, have shown significant therapeutic potential in reshaping the DNA methylation landscape of stromal and immune cells within the TME. As an example, Al-Kharashi et al. demonstrated that treatment with decitabine or eugenol reduced the proliferation, migration, and pro-tumoral effects of CAFs both in vitro and in vivo [156]. These compounds downregulated the expression of DNMT1 and DNMT3A at both the mRNA and protein levels in breast CAFs via E2F1 inactivation. Likewise, the DNMT inhibitor 5-Aza disrupted the epigenetic reprogramming of CAFs in an inflammatory milieu, restoring the normal fibroblast phenotype in vivo [78]. These findings suggest that DNMTi can alter methylation patterns in active CAFs and restrain their pro-tumor phenotypes. DNMTi could also reverse the immunosuppressive landscape via the repolarization of TAMs in the TME [157].

In addition, DNMTi has demonstrated potential in preventing MDSC accumulation in mouse models and cancer patients. Decitabine treatment prompted MDSCs to produce TNF-α, leading to RIP1-dependent necroptosis and consequently reduced MDSC accumulation in colorectal tumor-bearing mice [174]. In support of this, guadecitabine inhibited the systemic accumulation of MDSCs in a murine breast tumor model, enhancing the efficacy of adaptive immunotherapy by curbing MDSC proliferation and their immunosuppressive characteristics [121]. In an ongoing clinical trial, the combination of guadecitabine with immune checkpoint inhibitors (ICIs) showed durable clinical benefits in certain ovarian cancer patients [175]. In these patients, the combined treatment caused global hypomethylation in immune cells and the activation of antitumor immunity, as characterized by increased CD4^+^ T cells and classical monocytes in tumors. DNMT inhibition can also contract the de novo DNA methylation responsible for transforming effector T cells into exhausted T cells. By blocking this process, DNMTi, when combined with the PD-1 blockade, rejuvenates CD8^+^ T cells, leading to a synergized anti-tumor effect by preventing CD8^+^ T cell exhaustion [176]. Moreover, DNMT inhibition triggers antiviral defense mechanisms in both tumor and immune cells, boosting type I interferon signaling, remodeling the suppressive immune environment, and enhancing the efficacy of immune checkpoint blockade [177].

### 5.2. Histone Modification Inhibitors

While histone modification inhibitors are actively evaluated in clinical trials, their effects on reshaping the TME in preclinical models remain controversial. EZH2 inhibitors, for instance, have been shown to boost the antitumor immune response by activating the expression of immunostimulatory factors, including type I IFN genes [178], antigen-presenting machinery [179,180], and STING [181]. EZH2 is critical for the maintenance of regulatory T cell identity following activation [158]. The EZH2 inhibitor CPI-1205 reprograms tumor-infiltrating Tregs into a pro-inflammatory Th1-like phenotype [159]. The EZH2 inhibitor DZNep exerts a similar effect on Tregs, promoting an adaptive antitumor immune response in nasopharyngeal cancer [160]. However, contradictory observations have arisen regarding the effects of EZH2 inhibition. The EZH2 inhibitor GSK126 was reported to direct myeloid differentiation and resulted in MDSC accumulation and TIL exclusion in syngeneic mouse models of lung cancers and colon cancer [161]. Importantly, the methyltransferase activity of EZH2 is required for functional effector T cells according to a previous report [182]. EZH2 activates the Notch signaling pathway and stimulates the secretion of effector cytokines that promote the survival of tumor-infiltrating CD8^+^ T cells. Thus, the EZH2 inhibitor could inadvertently suppress the function of effector T cells.

Similarly, conflicting findings surround the impact of HDAC inhibitors on the TME in preclinical models. Scriptaid, an HDAC inhibitor, suppresses the conversion of endothelial cells into CAFs, thereby impeding CAF-driven tumor growth in vivo [162]. Other HDAC inhibitors have also been reported to inhibit CAF activation and tumor growth [163,164]. A low dose of adjuvant entinostat, combined with Aza, prevents cancer metastasis by inhibiting MDSC recruitment and disrupting premetastatic niche formation in lung and breast cancer models [165]. Other studies also demonstrate the suppression of MDSC accumulation and the establishment of immunosuppressive phenotypes by HDAC inhibitors [166,167,168]. However, earlier studies also suggest pro-tumoral microenvironmental changes upon HDAC inhibition [169,170].

### 5.3. Chromatin Remodeler Modulators

Small molecules that selectively target chromatin remodelers are rare, and the studies on their efficacy in reshaping TME are limited. For instance, the BET bromodomain inhibitor JQ1 showed synthetically lethal effects with the HDAC6 inhibitor ricolinostat in preclinical models of lung cancer [183]. This combination therapy enhanced MHC-II expression in tumor-infiltrating myeloid cells and acted in an NK cell-dependent manner. Later, the same group reported that JQ1 treatment inhibited the suppressive function of Tregs and therefore synergized with HDAC inhibition in lung cancer [171]. JQ1 treatment was also shown to reduce pancreatic tumor growth by suppressing the tumor-promoting effects of CAFs [172]. The BET inhibitor CPI203 yields similar effects on the stromal content of pancreatic cancer models, likely by blocking the GLI-SHH paracrine loop [173].

## 6. Conclusions

There are several reasons why caution must be exercised when applying epigenetic therapies in real clinical practice. First of all, epigenetic reagents typically target a similar catalytic domain rather than a specific protein, which may result in inhibitory effects on multiple proteins with distinct functions. Additionally, the inadvertent targeting of epigenetic agents to irrelevant proteins is not uncommon. For instance, 5-Aza has been found to bind directly to the cholesterol transporter ABCA9, reprogramming TAMs through a DNA methylation-independent mechanism [184]. It is even more complicated when considering the non-canonical functions of epigenetic proteins. For example, the enzymatic and non-enzymatic activities of the EZH2 protein have been reported to exert contrasting regulatory effects on TAM polarization [185]. Moreover, histone modification enzymes are known to catalyze lysine methylation or acetylation not only on histone proteins but also on non-histone proteins, thereby adding an additional layer of regulating mechanisms within TME [27,186]. Lastly, while a solitary epigenetic modification can exert certain functions, the transcriptional regulation of a particular gene often involves combinational or sequential modifications on local chromatin and distant regulatory elements. This highlights the necessity of evaluating the impact of combined epigenetic agents for effective cancer treatment.

**Figure 2:**
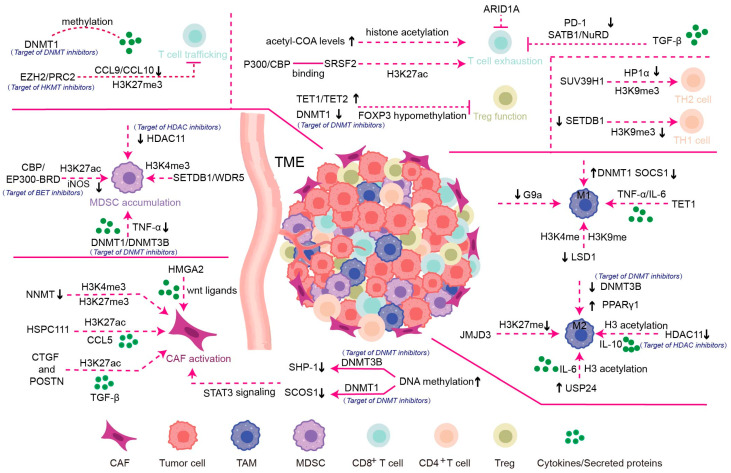
The main mechanisms by which epigenetic modulations remodel the TME. Aberrant epigenetic modulations of relevant genes in stroma and immune cells have diverse effects in reframing the TME. For instance, LIF increases DNMT3B expression to hypermethylate the *Shp-1* promoter [78], thereby activating and maintaining the JAK1/STAT3 pathway via DNMT1 [80]. Proteins such as NNMT [82], CTGF [85], and HSPC111 [86] regulate CAF activation through histone modifications. Epigenetic factors, including DNMT3B [92], DNMT1 [93], TET1 [95,96], JMJD3 [102,103], LSD1 [108,109,110], and HDAC11 [114], reprogram macrophages by enhancing the mRNA expression of PPARγ1 and promoting cytokine secretion. Additionally, DNMT1 [120], DNMT3B [121], CBP [123,124,125], SETDB1 [126] and HDAC11 [127] control MDSC accumulation. Together, DNMT1 and EZH2 suppress Th1-type chemokines CXCL9 and CXCL10 [131]. In Tregs, TET1 and TET2 convert 5mC to 5hmC in the *Foxp3* gene promoter, ensuring its hypomethylation and functional integrity [134,135]. SUV39H1 and SETDB1 regulate T cell differentiation through H3K9me3 modifications [141,142]. Furthermore, The P300/CBP complex, interacting with SRSF2, repositions acetylated H3K27 marks near immune checkpoint genes, which affects STAT3 recruitment and leads to T cell exhaustion [143]. The fluctuations in cellular acetyl-CoA levels, the main substrate for histone acetyltransferases, influence histone acetylation, which in turn impact TIL exhaustion [145,146]. Chromatin-remodelers such as NuRD complex [147] and ARID1A [148] also regulate TILs’ function. For detailed information on these mechanisms, see the references provided.

Despite the aforementioned limitations, epigenetic enzymes hold significant promise as targets of novel cancer therapies. Single-cell omics technology has revolutionized our ability to identify and characterize distinct cellular components, as well as comprehensively analyze the evolving dynamics of tumor microenvironments. In this review, we have summarized the well-established knowledge and recent advancements concerning the epigenetic regulation of major cellular components within tumors (Figure 2). The progress of cutting-edge technologies and innovative preclinical models is poised to deepen our understanding of the TME, as well as the spatiotemporal patterns of the epigenome, which could yield novel insights into the epigenetic regulation of the TME, potentially leading to optimized epigenetic therapies for cancer treatment. For example, the Assay for Transposase-Accessible Chromatin using sequencing (ATAC-seq) provides valuable insights into the TME by mapping accessible chromatin regions, which are essential for understanding epigenetic regulation in cancer [187]. By revealing how epigenetic modifications affect cellular behavior and communication between tumor and stromal cells, ATAC-seq helps identify potential therapeutic targets [188]. This technique enhances the ability to develop targeted epigenetic therapies by elucidating the intricate relationship between chromatin structure and gene regulation within the TME, ultimately paving the way for more effective and personalized cancer treatments [189,190]. Given the potent impact of epigenetic agents in normalizing the tumor immune microenvironment, we can anticipate the emergence of innovative therapeutic paradigms that harness the synergistic potential of epigenetic agents and immunotherapies, propelling cancer treatment to new heights.

## Figures and Tables

**Table 1:** Celular elements of TME.

Cell Type	Major Markers	Function	Reference
CAFs	α-SMA, S100A4, FAP, PDGFRα/β	Enhance tumor cell survival and growth; In specific contexts, CAFs counteract tumor progression by promoting anticancer immunity and regulating tumor-inhibitory signaling.	[39,40,41,42,43]
TAMs	CD68, CD163, ARG1, CD11b	Promote angiogenesis, metastasis andimmune evasion: Phagocytosis of cancer cells exerts an anti-tumor effect.	[44,45,46,47,48,49]
MDSCs	CD11b, Lin-, CD33, Gr-1	Suppress T-cell activity; Secrete immunosuppressive cytokines; Facilitate tumor progression.	[50,51,52,53,54,55,56]
CD8^+^ T	CD8, CD28, CD3, TCR, Tim 3, PD-1	Release perforin and granzymes; Secrete cytokines like lFN-γ, TNF-α; Kill cancer cells.	[57,58]
CD4^+^ T	CD4, CD28, CD3, TCR	Th1 subtype promotes the anti-tumoral response; TH2 subtype exerts the pro-tumoral effect.	[59,60]
Treg cells	CD4, CD25, FOXP3	Suppress the activity of effector T cells; Expediate tumor progression.	[61,62]
NK cells	CD56, CD16, KIRs, CD94	Recognize and kill tumor cells directly.	[63,64]
B cells	CD19, CD20, CD22, CD27	Drive the humoral immunity; Promote the anti-tumoral response.	[65,66]

**Table 2:** Epigenetic agents target TME.

Epigenetic Inhibitors	Target	Cell Type Within TME	Reference
**DNMT inhibitors**			
Decitabine+eugenol	DNMT1/DNMT3A	CAFS	[78]
Guadecitabine	DNMT1	MDSCs	[121]
Decitabine	DNMT1/DNMT3B	CAFS/MDSCs	[156]
5-AZA	DNMTS	CAFS/TAMs	[157]
**HKMT inhibitors**			
CPI-1205	EZH2	Tregs	[158,159]
Dzenp	EZH2	Tregs	[160]
GSK126	EZH2	MDSCs	[161]
**HDAC inhibitors**			
Scriptaid	HDAC1/3/8	CAFS	[162]
CUDC-907	Pan-HDAC	CAFS	[163,164]
Entinostat	Pan-HDAC	MDSCs/Tregs	[165]
SAHA	HDAC1/2/3	CAFS/MDSCs	[166,167]
CG-745	Pan-HDAC	MDSCS/TAMS	[168]
VPA	HDAC1	MDSCs	[169,170]
**BET inhibitors**			
JQ1	BRD4	CAFs/Tregs	[171,172]
CPI203	BRD2/3	Tregs	[173]

## Data Availability

No new data were created or analyzed in this study.
